# Polycyclic Aromatic Hydrocarbons Through the One Health Lens: Integrating Human, Animal, and Environmental Health Perspectives

**DOI:** 10.3390/toxics14050417

**Published:** 2026-05-11

**Authors:** Jose L. Domingo, Marília Cristina Oliveira Souza, Fernando Barbosa

**Affiliations:** 1 Laboratory of Toxicology and Environmental Health, School of Medicine, Universitat Rovira i Virgili, Sant Llorenç 21, 43201 Reus, Catalonia, Spain; 2 Department of Biomolecular Sciences, School of Pharmaceutical Sciences of Ribeirão Preto, University of São Paulo, Av. do Café s/n, Ribeirão Preto 14040-903, SP, Brazil; mcosouza@usp.br; 3 Department of Clinical, Toxicological and Bromatological Analysis, School of Pharmaceutical Sciences of Ribeirão Preto, University of São Paulo, Ribeirão Preto 14040-903, SP, Brazil; fbarbosa@fcfrp.usp.br

**Keywords:** combustion-derived contaminants, aryl hydrocarbon receptor, carcinogenicity, trophic transfer, biomonitoring

## Abstract

Polycyclic aromatic hydrocarbons (PAHs) are ubiquitous combustion-derived contaminants that represent a significant cross-cutting threat to human, animal, and environmental health. Viewed through an explicit One Health lens, this review shows how the shared combustion sources, evolutionarily conserved toxicological mechanisms, and food-web linkages connecting environmental contamination to wildlife and human exposure justify an integrated, cross-domain approach to PAH risk assessment and management. PAHs are generated predominantly through incomplete combustion of organic materials and are globally distributed through atmospheric transport, aquatic runoff, and food-web transfer, persisting in soils and sediments for decades. The present review synthesizes current knowledge on PAHs through an explicit One Health lens, examining shared sources, environmental fate, and convergent health effects across species and health domains, while also highlighting the need to move beyond the classical US EPA priority PAHs to include high-molecular-weight PAHs (>302 Da), alkylated homologues, and transformation products such as oxy- and nitro-PAHs. Common pathways such as dietary intake of grilled and smoked foods, inhalation of contaminated air, and occupational exposure create parallel toxicological burdens in both human and wildlife populations, particularly through genotoxic mechanisms mediated by aryl hydrocarbon receptor (AhR) activation and CYP1A1/CYP1B1-catalyzed bioactivation to reactive diol epoxides. The resulting DNA adduct formation links environmental PAH exposure to carcinogenicity, reproductive toxicity, immunosuppression, and developmental impairment across vertebrate species with remarkable mechanistic consistency. Wildlife, especially fish, marine mammals, and seabirds, serve as critical sentinels for environmental PAH contamination, while simultaneously facing direct health impacts on immune function, reproduction, and population viability. Vulnerable human populations, including children, subsistence communities, occupational workers, and residents near combustion-intensive industries, bear disproportionate burdens reflecting underlying environmental justice concerns. Integrated intervention strategies encompassing source control, dietary exposure reduction, site remediation, and coordinated biomonitoring are urgently needed. By incorporating emerging PAH classes with distinct persistence, trophic behavior, and toxicological potency, the One Health paradigm provides a more comprehensive conceptual framework for modern environmental surveillance, food safety, and integrated risk assessment, recognizing that the health of terrestrial and aquatic ecosystems is inseparable from that of the animals and humans within them.

## 1. Introduction

### 1.1. Polycyclic Aromatic Hydrocarbons

Polycyclic aromatic hydrocarbons (PAHs) constitute one of the largest and most chemically diverse families of environmental pollutants. PAHs comprise hundreds of compounds that are formed mainly through the incomplete combustion of organic materials and the thermogenic transformation of fossil fuels [1,2,3]. These molecules are characterized by two or more fused benzene rings arranged in planar or angular configurations, a structural feature that underlies both their chemical stability and their potent biological activity [4,5]. The United States Environmental Protection Agency (US EPA) originally designated 16 PAHs as priority pollutants based on their environmental prevalence, persistence, and toxicological significance, with benzo[a]pyrene (BaP) widely recognized as the prototypical representative and classified as a Group 1 human carcinogen by the International Agency for Research on Cancer [6]. Previously, the EFSA Panel on Contaminants adopted an opinion on suitable indicators for the occurrence and toxicity of PAHs in food. It was concluded that BaP, the only PAH presently regulated in food, was not a suitable indicator for the occurrence of PAHs in food. Therefore, to enhance consumer health protection, it has been recommended to use a set of four or eight representative PAHs as more suitable indicators for evaluating food safety risks, recognizing that combined exposure assessments more accurately represent real-world contamination patterns [7].

Although the 16 priority PAHs remain central to regulatory frameworks, contemporary analytical and toxicological evidence indicates that this list captures only part of the real-world PAH exposome. High-molecular-weight PAHs (>302 Da), alkylated congeners, and secondary transformation products have increasingly been associated with enhanced persistence, selective food-web transfer, and in some cases greater mutagenic or oxidative potential. Consequently, a modern One Health interpretation requires expanding beyond legacy target lists toward broader congener-resolved surveillance. The atmospheric concentrations of PAHs vary markedly depending on the environmental context. In urban settings, total PAH concentrations in ambient air typically range from 10 to over 100 ng/m^3^, with BaP concentrations frequently exceeding the WHO guideline value of 1 ng/m^3^ in cities across Asia and Eastern Europe, while in rural areas concentrations are generally one to two orders of magnitude lower [8,9]. In aquatic sediments, ΣPAHs commonly range from tens to thousands of μg/kg dry weight in contaminated harbors and industrial zones, with BaP concentrations in severely impacted sites reported up to 500 μg/kg dry weight [10]. These concentration gradients across environmental media highlight the need for context-specific risk governance.

PAH contamination is essentially a universal consequence of combustion, spanning sources as varied as forest fires and volcanic activity to motor vehicle exhaust, industrial processes, residential heating, and the cooking of food [8,9,11]. Once released into the environment, PAHs associate strongly with particulate matter and organic carbon, facilitating long-range atmospheric transport and subsequent deposition across the globe, including remote polar regions and high-altitude ecosystems [12,13]. Their hydrophobic and lipophilic character promotes partitioning into sediments and organic-rich soils, where they may persist for decades, creating reservoirs of long-term contamination that continue to expose organisms long after source activities have ceased [4,14].

The toxicological importance of PAHs mainly depends on their metabolic activation [15]. In mammals, PAHs are activated and metabolized by cytochrome P450 enzymes, especially CYP1A1, CYP1A2, and CYP1B1, which are triggered by the aryl hydrocarbon receptor (AhR) pathway, yielding reactive diol epoxide intermediates that can form covalent bonds with DNA [16,17]. Benzo[a]pyrene-7,8-diol-9,10-epoxide (BPDE), the final carcinogenic metabolite produced during BaP metabolism, is one of the most well-understood genotoxic intermediates. It forms covalent DNA adducts that lead to specific mutations in oncogenes and tumor suppressor genes, providing a mechanistic connection between PAH exposure and cancer development in both experimental studies and human epidemiological research [18,19,20]. In addition to carcinogenic effects, PAHs can cause immunotoxicity, endocrine disruption, reproductive and developmental harm, and neurotoxicity, effects observed across various species [2,4,21].

### 1.2. The Case for a One Health Approach

The One Health concept recognizes the fundamental, inextricable interconnection among human, animal, and environmental health, providing an essential framework for understanding and responding to complex, multi-domain environmental contamination challenges [22,23]. This approach, which has evolved from its origins in veterinary medicine and comparative medicine to encompass broader ecological and planetary health concerns, is particularly well suited to addressing the PAH challenge [24,25,26].

Several intrinsic features of PAH contamination make it an exemplary case for the One Health application. First, the combustion sources generating PAHs operate in the same environments where human and animal populations live, creating shared atmospheric exposure pathways with no meaningful ecological boundary. Second, food web transfer, mainly through aquatic ecosystems, where PAHs accumulate in sediments and benthic organisms, creates direct linkages between ecosystem contamination and the dietary exposure of both wildlife and humans who consume fish and seafood. Third, and perhaps most compelling from a mechanistic standpoint, the AhR-mediated toxicological pathway responsible for PAH carcinogenicity is among the most evolutionarily conserved signaling axes in vertebrate biology [27,28,29]. This convergence of exposure and mechanism across species positions PAHs as a paradigmatic case for cross-species toxicological integration.

Recently, we have applied One Health frameworks to other environmental pollutants, including polybrominated diphenyl ethers (PBDEs) [30], polychlorinated naphthalenes (PCNs) [31], and dioxins and furans (PCDD/Fs) [32]. The present review extends this analytical approach to PAHs, a contaminant class that, despite decades of intensive research, has not previously been systematically synthesized through an explicit One Health lens.

### 1.3. Objectives

**Figure 1:**
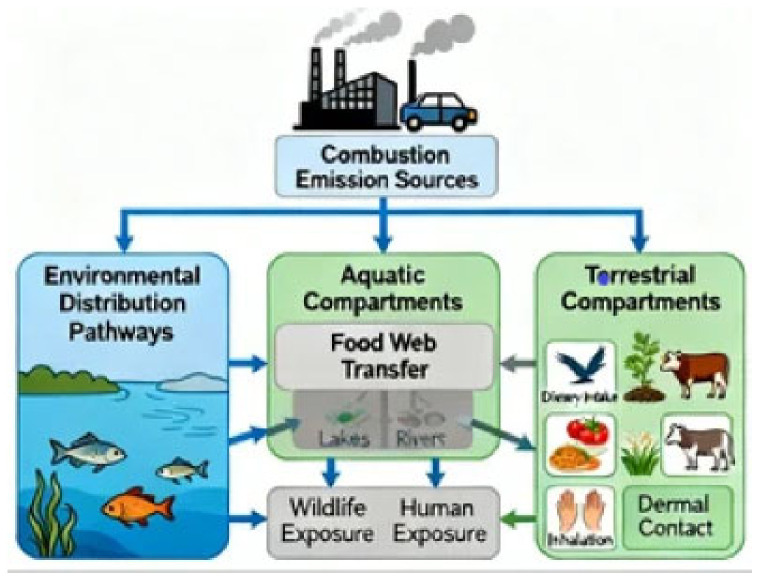
Conceptual One Health framework for PAH contamination. Arrows represent (i) combustion emission sources, (ii) environmental distribution and food web transfer pathways through aquatic and terrestrial compartments, and (iii) convergent exposure routes to wildlife and human populations (dietary intake, inhalation, dermal contact).

This review aims to synthesize current knowledge on PAH contamination through an explicit One Health framework, examining interconnected sources, environmental fate, exposure pathways, and health effects across human, animal, and environmental domains. The specific objectives were: (1) to characterize shared sources and environmental fate processes creating global PAH contamination patterns, (2) to evaluate convergent health impacts across human and wildlife populations, (3) to identify critical interconnections and shared vulnerabilities linking the three health domains, (4) to analyze environmental justice considerations in PAH exposure, with attention to vulnerable groups including subsistence communities and occupational workers, (5) to propose integrated intervention strategies grounded in One Health principles, and (6) to highlight knowledge gaps requiring transdisciplinary collaboration. The conceptual framework is depicted in Figure 1, which illustrates the interconnected emission sources, environmental distribution pathways, food web transfer routes, and convergent exposure to both wildlife and human populations. This integrated perspective is essential for addressing the multigenerational and transboundary dimensions of persistent organic pollutant contamination [26,30].

## 2. Methods

### Search Strategy

A review of the scientific literature was conducted to synthesize evidence on PAHs within an explicit One Health framework, covering publications through 28 February 2026. Databases consulted were PubMed, Scopus, Web of Science, and Google Scholar. Search terms and their combinations were employed using Boolean operators (AND, OR): “polycyclic aromatic hydrocarbons” OR “PAHs” OR “benzo[a]pyrene” OR “PAH mixture”; “One Health” OR “EcoHealth” OR “environmental health”; “bioaccumulation” OR “biomagnification” OR “food web”; “exposure pathway” OR “human exposure” OR “wildlife exposure” OR “dietary exposure”; “aryl hydrocarbon receptor” OR “AhR” OR “CYP1A1” OR “DNA adducts”; “carcinogenicity” OR “genotoxicity” OR “immunotoxicity” OR “reproductive toxicity” OR “developmental toxicity”; “fish” OR “marine mammals” OR “seabirds” OR “wildlife”; “risk assessment” OR “regulation” OR “remediation”; “environmental justice” OR “vulnerable populations” OR “occupational exposure”.

The included studies were those focused on PAHs or specific PAH compounds, demonstrating clear relevance to at least two domains of the One Health triad (human, animal, environment), and reported original data, systematic reviews, meta-analyses, or significant conceptual advances. Literature from international organizations (IARC, EFSA, ATSDR, US EPA, WHO) was also consulted. The selected literature was analyzed through a structured One Health framework identifying: (a) shared sources and exposure pathways, (b) convergent toxicological mechanisms and health effects, (c) feedback loops and interconnections between health domains, (d) vulnerable populations and environmental justice considerations, (e) integrated intervention strategies with co-benefits across domains, and (f) critical knowledge gaps requiring transdisciplinary research.

## 3. Results

### 3.1. PAH Sources, Environmental Fate, and Shared Exposure Pathways

#### 3.1.1. Combustion Sources and Environmental Release

PAH contamination originates predominantly from high-temperature, incomplete combustion of organic materials, with the relative contribution of specific sources varying considerably across geographic settings and levels of industrialization [9,15,33]. Mobile sources, particularly gasoline- and diesel-powered vehicles, are the dominant contributors to urban air pollution, with diesel exhaust particles serving as particularly significant carriers of high-molecular-weight PAHs [2,34]. Industrial combustion processes, including coke production, aluminum smelting, coal gasification, and petroleum refining, generate large point-source emissions that create hotspots of localized contamination and contribute to regional atmospheric burdens through stack emissions [4,9]. Moreover, biomass burning, encompassing both deliberate agricultural burning and wildfires, is quantitatively one of the largest global PAH sources, with increasing significance under scenarios of heightened wildfire frequency linked to climate change [11,35].

Residential and indoor combustion sources such as coal and wood heating, tobacco smoking, and the cooking of food at high temperatures generate PAH exposures that may substantially exceed those from ambient outdoor air in poorly ventilated settings, especially in low-income households in Asia, Africa, and parts of Latin America [2,36]. Grilling, charbroiling, and smoking of meat, fish, and other protein-rich foods at elevated temperatures produce PAHs through pyrolysis of organic macromolecules and direct deposition of combustion gases onto food surfaces [7,37,38]. Thus, dietary intake of thermally processed foods represents not merely a food safety concern, but a PAH exposure pathway inextricably linked to the combustion chemistry that drives atmospheric and environmental contamination. Among the atmospheric and indoor sources, it is important to note that nitro-PAHs (NPAHs) and oxygenated PAHs (OPAHs), the transformation products of parent PAHs, may reach total concentrations exceeding those of the parent PAHs themselves in PM2.5. Studies conducted in Southern European cities have reported that ΣNPAHs and ΣOPAHs in PM2.5 surpassed ΣPAHs levels, while OPAHs and NPAHs together contributed substantially to the estimated lifetime excess cancer risk at all monitored sites [39].

Natural sources such as volcanic eruptions, forest fires, and biosynthesis by certain microorganisms contribute to a background level of PAHs [15,40]. During wildfires, large quantities of PAHs, especially compounds like phenanthrene, fluoranthene, and pyrene, are released into the atmosphere in both gaseous and particulate phases. Although they are minor in comparison to anthropogenic emissions in most inhabited regions, they confirm the pre-industrial ubiquity of these compounds and the evolutionary context in which PAH-metabolizing enzymes arose [4,15,40].

#### 3.1.2. Environmental Fate and Persistence

Once released into the environment, PAHs undergo complex partitioning between atmospheric, aquatic, and terrestrial compartments governed by their physicochemical properties, particularly molecular weight, octanol–water partition coefficient (log Kow), and vapor pressure [4,41]. The physicochemical properties of individual PAH compounds directly govern their environmental partitioning and fate. Lower-molecular-weight PAHs (2–3 rings, e.g., naphthalene, fluorene) exhibit higher vapor pressures and water solubility, remaining mobile in atmospheric and aquatic phases. Naphthalene, for example, has a log Kow of 3.37, a vapor pressure of ~1 Pa at 25 °C, and water solubility of ~31 mg/L, while higher-molecular-weight compounds (4–6+ rings, e.g., benzo[a]pyrene, dibenzo[a,h]anthracene) are more strongly associated with particulate matter, sediment organic carbon, and biological lipids. BaP has a log Kow of 6.04, a vapor pressure of ~7 × 10^−7^ Pa at 25 °C, and water solubility of only ~3.8 μg/L [33,42].

In aquatic environments, the strong hydrophobicity of PAHs causes them to partition primarily to suspended particles and bottom sediments, where their concentrations are typically 2–4 orders of magnitude higher than in the overlying water column [4,10]. Sediment contamination is particularly persistent, as burial below the aerobic/anaerobic interface protects PAHs from photodegradation and limits microbial access [43]. Urban stormwater runoff transporting road-associated PAHs also constitutes a major and often underappreciated pathway for aquatic contamination [44]. Long-range atmospheric transport of particle-bound PAHs enables deposition in remote polar and alpine environments, where they have been detected in Arctic marine sediments, alpine lake sediments, and even the cryosphere, raising concerns about their entry into pristine food webs [45].

Abiotic degradation of PAHs in surface environments occurs primarily through photodegradation, producing oxygenated metabolites that may retain or even exceed the toxicity of the parent compounds [4,41]. Microbial biodegradation of PAHs by soil and sediment bacteria, particularly species of *Sphingomonas*, *Mycobacterium*, and *Rhodococcus*, constitutes the principal natural attenuation mechanism in contaminated environments. However, degradation rates vary widely and are generally most effective for low-molecular-weight PAHs when environmental conditions (e.g., oxygen, nutrients and PAH bioavailability) are favorable [46,47,48]. High-molecular-weight PAHs relevant to human carcinogenicity risk, including BaP and dibenzo[a,h]anthracene, are substantially more recalcitrant to biodegradation, contributing to their persistence in contaminated soils and sediments for decades following source elimination [15,49]. The interrelationship between PAH concentrations across environmental matrices (air, soil, water, and sediment) is governed by these same physicochemical parameters, with fugacity modeling demonstrating that PAH levels in soil are often correlated with those in air at urban sites [50].

#### 3.1.3. Food Web Transfer and Bioaccumulation

Beyond the historically prioritized 16 PAHs, future trophodynamic studies should explicitly incorporate high-molecular-weight PAHs (>302 Da), alkylated homologues, and transformation products such as oxy- and nitro-PAHs. These compounds may exhibit distinct retention patterns, selective trophic transfer, and toxicological relevance that are not adequately captured by traditional assessments focused on the US EPA priority PAHs. In addition, apparent trophic dilution of parent PAHs should not preclude the possibility of selective retention in benthic organisms, tissue-specific accumulation, or transfer of reactive metabolites along aquatic food webs [42].

The transfer of PAHs through food webs is mechanistically distinct from the biomagnification classically observed for organochlorine pollutants such as PCBs and PCDD/Fs, owing to the capacity of most organisms above the lowest trophic levels to metabolize PAHs through CYP1A-mediated pathways [21,51,52]. Consequently, the general pattern observed in aquatic food webs is one of trophic dilution, in which PAH concentrations in tissues decrease at higher trophic levels relative to lower trophic levels and ambient sediment/water concentrations [53,54]. This metabolic capacity, while protective against PAH accumulation, generates reactive metabolites responsible for the genotoxic and carcinogenic effects that make PAHs a public health concern, and is fundamental to understanding their toxicology [55]. In this context, petroleum hydrocarbons, including alkanes, naphthenes, and aromatic fractions, frequently co-occur with PAHs in contaminated environments, particularly in the aftermath of oil spills or in petrochemical industrial zones. They may modulate PAH fate and toxicity through competitive CYP1A induction and altered bioavailability [56].

Nevertheless, benthic and filter-feeding invertebrates that lack the vertebrate CYP1A metabolic pathway, including mussels, clams, and polychaete worms, bioconcentrate parent PAHs from contaminated sediments to high levels, serving as effective integrating sentinels of aquatic PAH contamination [51]. Fish accumulate PAHs primarily from water and sediment through gill absorption and dietary ingestion, with the liver as both the primary site of CYP1A-mediated metabolism and a locus of resulting hepatic pathology [57,58]. The formation of PAH-DNA adducts in fish liver tissue, mainly in flatfish species inhabiting contaminated urban harbor sediments, has been reported and serves as a biomarker of biologically effective dose [59,60].

Despite their elevated metabolic capacity, marine mammals and seabirds accumulate PAH metabolites in bile and tissues, being exposed through both dietary intake from contaminated prey and inhalation of petroleum-associated vapors following oil spills [61,62]. Major oil spill events have provided tragic but scientifically informative case studies of acute and chronic PAH exposure impacts on marine wildlife populations, reporting effects ranging from acute hepatotoxicity and respiratory damage to long-term reproductive suppression in affected populations [63,64,65]. To synthesize these cross-domain linkages in a single overview, Table 1 summarizes the major PAH sources, exposure pathways, adverse effects, and key interconnections across environmental, wildlife, and human health domains.

### 3.2. Human Health Impacts: Pathways and Effects

#### 3.2.1. Exposure Pathways

Human exposure to PAHs occurs through multiple interconnected pathways, with dietary intake generally recognized as the primary route for the non-smoking, non-occupationally exposed general population [7,38,66,67]. These human exposure routes and health endpoints are situated within the broader One Health context, summarized in Table 1. Foods that have been grilled, charbroiled, barbecued, smoked, or subjected to high-temperature cooking, with direct contact with flames or combustion gases, contain the highest PAH concentrations, particularly of high-molecular-weight carcinogenic compounds including BaP and benzo[a]anthracene [37]. Grilled meats and smoked fish have been identified as the dominant contributors to PAH dietary intake across European, American, African, and Asian dietary surveys [7,67,68]. Regulatory maximum levels for BaP in smoked and grilled meat products in the European Union stand at 2 μg/kg fresh weight, while the PAH4 sum (BaP, chrysene, benz[a]anthracene, benzo[b]fluoranthene) is established at 12 μg/kg. In the dietary exposure studies reviewed, BaP concentrations in grilled meat and smoked fish samples ranged from <0.1 to over 50 μg/kg, with high-temperature charbroiled products most likely to exceed regulatory thresholds [7,38]. Although EU regulatory limits for BaP and PAH4 provide useful safety benchmarks, they are based on a limited set of PAHs and assume simple additive effects. However, real-world exposure involves more complex mixtures, including high-molecular-weight, alkylated, and oxygenated/nitrated PAHs, some of which may be equally or more toxic. This suggests current frameworks may underestimate cumulative risk, particularly from a One Health perspective integrating human, animal, and environmental exposures.

Inhalation represents the second major exposure pathway, with ambient air PAH concentrations varying enormously between urban and rural environments, and between industrialized and residential areas [2,69]. Urban dwellers, particularly those residing near heavily trafficked roads, petrochemical facilities, or coal-burning power plants, face substantially higher inhalation exposures than their rural counterparts [34,70]. Indoor air quality may be equally or more important than outdoor exposure in settings where solid fuels (coal, wood, dung) are burned for cooking and heating, conditions affecting hundreds of millions of people globally and generating PAH-laden air that substantially exceeds WHO guideline values for BaP [2,9].

Occupational exposure represents the highest magnitude exposure scenario for specific worker populations, with elevated PAH burdens found in coke oven workers, chimney sweeps, asphalt and road paving workers, aluminum smelter workers, firefighters, and petroleum refinery employees [41,71,72,73]. Coke oven workers have historically provided some of the strongest evidence linking PAH exposure to lung cancer through occupational epidemiology, while contemporary firefighter cohorts have also emerged as an important high-exposure population in developed nations, where PAH-laden combustion products from structural fires penetrate protective equipment [41,72,74]. Wildland firefighters experience intense occupational exposure to PAHs primarily through inhalation of smoke and, to a lesser extent, dermal absorption during fire suppression activities. This exposure is particularly critical because wildfire smoke is a complex mixture containing particulate matter, volatile organic compounds, and PAHs adsorbed onto fine particles (PM2.5), which facilitates deep lung deposition [40]. Urinary 1-hydroxypyrene (1-OHP) serves as the principal validated biomarker of recent PAH exposure, being recommended as the standard monitoring tool for occupationally exposed workers, with Biological Exposure Indices (BEI) set at 0.5 μmol/mol creatinine by the ACGIH [75].

Dermal absorption, especially through soil contact at contaminated sites [76] and from occupational handling of PAH-containing materials, such as coal tar products and creosote-treated wood, constitutes a significant but often underestimated exposure route [75,77]. Maternal transfer of PAHs across the placenta and through breast milk creates a critical exposure pathway during vulnerable developmental windows [78,79,80]. Epidemiological studies measuring PAH-DNA adducts in cord blood and infant DNA have shown fetal exposure with consequences for birth outcomes and neurodevelopment [81,82].

#### 3.2.2. Human Health Effects

The human health effects of PAH exposure are dominated by carcinogenicity, with lung cancer representing the most extensively reported endpoint in occupational epidemiology and the endpoint underlying regulatory standards for benzo[a]pyrene in air [6,83]. The molecular mechanism, AhR-mediated induction of CYP1A1 and CYP1B1, bioactivation of PAHs to reactive diol epoxides, covalent DNA adduct formation, and mutation at hotspot codons in tumor suppressor genes, including TP53, has been characterized with exceptional mechanistic resolution [16,18,19]. Benzo[a]pyrene preferentially induces G-to-T transversions at hotspot codons of TP53, particularly codons 157, 248, and 273, mirroring the mutation spectrum observed in lung cancers from smokers and occupationally exposed populations, and providing molecular epidemiological evidence that environmental PAH exposure contributes directly to human carcinogenesis [19,84,85].

Exposure to PAHs, along with other stressors, has been linked to early-life genetic damage, as well as the disruption of epigenetic development in metabolic, endocrine, and neural pathways. In this context, 8-hydroxy-2′-deoxyguanosine (8OHdG) is often used as a biomarker of oxidative stress-induced DNA damage from chemicals. This lesion results from the oxidation of guanine at the C8 position under pro-oxidative conditions. Despite its widespread use, the reliability of 8OHdG as an indicator of PAH-related genotoxicity remains uncertain. Evidence currently available is inconsistent and sometimes conflicting, likely due to differences in study design, exposure assessment, biological samples, and analytical techniques. These challenges emphasize the need for more rigorous and standardized methods to better understand the link between PAH exposure and oxidative DNA damage [18,19,20,80]. According to Ferreira-Azevedo et al. [86], high-molecular-weight PAH isomers differ substantially in their toxicological properties. Among them, dibenzo(a,l)fluoranthene exhibited the greatest potency, producing concentration-dependent cell toxicity, pronounced DNA lesions, and increased 8-OHdG levels. All isomers were able of inducing apoptosis and genotoxicity, emphasizing that molecular structure exerts a decisive influence on toxicity and that assessing PAHs in mixtures may underestimate actual health risks.

In addition to lung cancer, PAH exposure has also been associated with cancers of the bladder, skin, colorectum, and upper aerodigestive tract in occupationally exposed populations, and with childhood leukemia and other malignancies in those with elevated prenatal exposures [6,85,87]. The carcinogenic risk extends to ingested PAHs, with animal studies showing gastric and intestinal carcinogenesis following oral administration, a pathway relevant to human risk through smoked food consumption [7].

Reproductive and developmental toxicity represents a growing area of concern [88]. Benzo[a]pyrene disrupts the hypothalamic–pituitary–gonadal axis, induces oxidative stress in germ cells, and interferes with steroidogenesis through AhR-mediated anti-estrogenic activity [41]. Epidemiological studies have linked prenatal PAH exposure, assessed through maternal urinary PAH metabolites and cord blood adduct levels, to reduced birth weight, intrauterine growth restriction, preterm birth, and adverse neurodevelopmental outcomes, including cognitive deficits and behavioral problems in childhood [81,82].

On the other hand, immunotoxic effects of PAHs include suppression of both innate and adaptive immune responses, reported in both occupational cohorts and experimentally exposed rodents, with implications for increased susceptibility to infectious disease and impaired immune surveillance of nascent tumors [89]. Cardiovascular effects, including endothelial dysfunction and accelerated atherosclerosis, have been associated with PAH exposure through mechanisms involving oxidative stress and inflammatory signaling. This is consistent with the epidemiological observation of elevated cardiovascular mortality in occupationally exposed cohorts and in communities subject to high air PAH levels [71,90].

#### 3.2.3. Vulnerable Populations and Health Disparities

The distribution of PAH exposure burden across human populations is far from uniform, reflecting inequities in environmental contamination, occupational risk, dietary practices, and physiological susceptibility. Vulnerable populations such as pregnant women, children, and infants show distinct but worrisome PAH exposure profiles. In Brazilian pregnant women, PAH metabolites are almost universally detected, and carcinogenic risk estimates often exceed acceptable levels. For example, Cesila et al. [79] reported excess lifetime cancer risk estimates above 10^−4^ for BaP metabolites in a cohort of 90 pregnant women from Ribeirão Preto, Brazil, with hydroxypyrene detected in 100% of urinary samples. This vulnerability likely stems from physiological sensitivity and the capacity of PAHs to cross the placenta, posing threats to fetal growth and development. Children are also highly sensitive, with greater exposure associated with urban settings, diet, and environmental factors. Despite non-carcinogenic risk classifications, their developmental immaturity increases the likelihood of long-term health impacts. Conversely, infants generally present lower metabolite concentrations, presumably due to incomplete metabolic function. Children and developing fetuses represent populations of particular concern, which is due to their higher ventilation rates per unit body weight, greater absorptive capacity in the gastrointestinal tract, and the heightened sensitivity of developing organ systems (particularly the nervous system and immune system) to genotoxic and endocrine-disrupting insults during critical developmental windows [67,69,79,80,81,82].

Communities proximate to industrial PAH sources (coking operations, petroleum refineries, asphalt production facilities, and heavily trafficked urban corridors) experience disproportionate inhalation and soil-contact exposures [2]. These communities frequently include environmental justice populations, defined by their disproportionate proximity to pollution sources combined with reduced access to healthcare and political representation [91,92]. Epidemiological studies have found elevated PAH-DNA adduct burdens in residents of such communities, including children, providing direct biological evidence of the health toll exacted by environmental inequities [81]. Studies across different industrialization contexts, from sub-Saharan Africa to Southeast Asia, have demonstrated that communities in low- and middle-income settings commonly experience PAH exposures far exceeding those measured in Western populations subject to existing regulatory frameworks, underscoring the global inequity in PAH health risk burdens [67,93,94].

Subsistence communities relying heavily on smoked or traditionally fire-prepared foods, and those consuming fish from contaminated urban waterways, face dietary PAH exposures substantially exceeding those estimated for general Western populations. Some estimates for traditional smoked food-dependent communities suggest dietary BaP intakes 5–20 times the European Food Safety Authority tolerable intake benchmark [7,95]. This is particularly relevant for indigenous and low-income communities whose traditional food systems, central to cultural identity and nutritional security, may simultaneously serve as primary pathways for PAH exposure, presenting an environmental justice dilemma analogous to that posed by other organic pollutants.

Riverside populations are a socially vulnerable group with significant exposure to polycyclic aromatic hydrocarbons (PAHs), mainly due to environmental contamination and lifestyle factors. Human biomonitoring studies show that urinary PAH metabolites (OH-PAHs) are common in these communities, with naphthalene metabolites detected in 100% of samples and overall levels often exceeding those reported in other populations. This higher internal exposure is linked to local sources, including pyrogenic emissions, boat traffic, and consumption of contaminated aquatic organisms. Notably, urinary OH-PAHs are reliable markers of recent exposure, as they reflect the internal dose of PAHs through their quick metabolism and excretion. Risk assessments suggest possible non-cancer health effects and raise concerns about cancer risks, especially from benzo[a]pyrene metabolites, highlighting the importance of ongoing monitoring and public health efforts in these vulnerable communities [96]. Studies in school and preschool environments show a positive correlation between indoor air PAH concentrations and urinary PAH metabolite levels in children. Benzo[b]fluoranthene and pyrene were among the predominant species detected in school environments in both urban and semi-rural settings. It reinforces the importance of indoor air as a relevant exposure pathway for children beyond home [97]. Nursing mothers represent an additional vulnerable group. Breast milk PAH levels reflect maternal dietary and occupational exposures, with studies reporting PAH metabolites in breast milk with potential relevance for infant dose estimation, particularly in settings where solid fuel combustion predominates [78,80]. These findings reveal a key limitation in current risk assessments, which rely on single compounds or simple mixtures and average population exposures. Vulnerable groups, such as children, pregnant women, and subsistence communities, are often underrepresented. Therefore, guidelines should be updated using population-specific scenarios, biomonitoring data, and cumulative risk approaches that better reflect real-world exposures.

### 3.3. Wildlife Health Impacts: Sentinels and Shared Vulnerabilities

#### 3.3.1. Exposure Pathways and Bioaccumulation in Aquatic Species

Wildlife populations serve as both critical environmental sentinels and direct conservation concerns in the context of PAH contamination [51,62,98,99]. Aquatic organisms occupy the most extensively studied and toxicologically important position in the One Health PAH framework, given their direct exposure to contaminated sediments and their role as food web integrators connecting environmental contamination to human dietary exposure. Fish accumulate PAHs through multiple routes (gill absorption from the water column, ingestion of contaminated sediment particles, and consumption of contaminated benthic prey), with the relative importance of each pathway varying by species, life stage, and habitat [57,58]. Quantitatively, BaP concentrations in the bile of fish from contaminated harbor sediments have been reported at several orders of magnitude above those in the water column, reflecting the efficiency of CYP1A-mediated bioactivation and biliary excretion pathways. For example, English sole (*Parophrys vetulus*) from contaminated sites in Puget Sound (USA) showed hepatic PAH-DNA adduct levels of up to 50 pmol/mg DNA, compared to <5 pmol/mg DNA in fish from reference sites, directly linking sediment contamination to biologically effective dose [60].

Benthic invertebrates, including polychaete worms, amphipods, bivalves, and certain crustaceans, accumulate PAHs to concentrations substantially higher than the surrounding sediment through bioconcentration and, for those with limited metabolic capacity, minimal biotransformation [21,51]. Mussels (*Mytilus* spp.) and oysters (*Ostrea* and *Crassostrea* spp.) have been extensively deployed in environmental monitoring programs precisely because their limited PAH metabolism renders them reliable integrators of waterborne and sediment contamination [100,101]. Pacific herring and other small forage fish that consume benthic invertebrates and zooplankton accumulate PAHs in their tissues and represent the dietary source of PAH exposure for higher-trophic-level piscivores, including marine mammals and seabirds [58,65].

Marine mammals encounter PAHs through a combination of dietary ingestion, inhalation of petroleum vapors in contaminated surface waters and, particularly in nursing animals, maternal transfer. Oil spill events have provided the most acute and dramatic PAH exposures in wildlife, with the aftermath of the Deepwater Horizon blowout yielding extensive documentation of PAH effects in Atlantic bluefin tuna cardiac muscle, bottlenose dolphin respiratory disease, and Gulf sturgeon larval mortality [63,64]. The cardiac toxicity of PAH mixtures in developing fish embryos, mediated through AhR-independent disruption of potassium channel function in the heart, emerged as a key toxicological discovery from Deepwater Horizon research, expanding the understanding of PAH mechanisms beyond classic AhR-mediated genotoxicity [64].

#### 3.3.2. Wildlife Health Effects

Health impacts of PAH exposure observed in free-living wildlife closely resemble those described in experimental animal models and in humans, consistent with the strong evolutionary conservation of AhR signaling and shared cellular mechanisms of PAH-induced DNA damage [21,28,102]. Hepatotoxicity and genotoxicity are among the best-characterized effects in fish, with hepatic neoplasms, elevated CYP1A expression, and DNA adduct formation documented in flatfish populations inhabiting contaminated urban harbors including Boston Harbor, Puget Sound, and the Elizabeth River [60,103]. The prevalence of liver tumors in English sole (*Parophrys vetulus*) from heavily contaminated sites in Puget Sound provided foundational evidence linking environmental PAH exposure to carcinogenesis outside the laboratory [103]. Data from additional monitoring programs in European coastal waters have extended these findings. Thus, studies in flatfish from the Southern Baltic, North Sea estuaries, and Portuguese estuaries have reported elevated frequencies of hepatic lesions and increased CYP1A expression consistent with PAH exposure, which show that these sentinel endpoints are not geographically limited to North American hotspots [104,105].

Reproductive and developmental toxicity in fish includes reduced fertilization success, embryotoxicity, cardiac deformities, craniofacial malformations, and reduced survival of offspring from PAH-exposed parents [106]. These effects are largely attributable to the cardiac and craniofacial teratogenicity of specific high-molecular-weight PAH congeners acting through both AhR-dependent and AhR-independent pathways [57,64]. Immunosuppression, manifested as increased susceptibility to parasitism and bacterial infection, has been reported in PAH-exposed fish and constitutes a significant mechanism through which sublethal PAH contamination may affect population health [107].

In marine mammals, PAH exposure was associated with hepatocellular carcinoma in beluga whales (*Delphinapterus leucas*) from the heavily industrialized St. Lawrence Estuary, where a unique tumor syndrome linked to both PAH body burden and dietary exposure has been extensively characterized over several decades [62]. These cancer-ridden belugas serve as a powerful emblem of the One Health principle: the same PAH contamination driving cancer in an industrial waterway predicts elevated cancer risk in the human communities sharing the same food web and air shed.

On the other hand, seabirds accumulate PAH metabolites primarily through dietary exposure to contaminated fish and crustaceans [108]. Oil spills cause catastrophic acute mortality through hypothermia resulting from feather oiling, aspiration of petroleum vapors, and gastrointestinal toxicity from ingested oil, but sublethal exposure to PAH mixtures during chronic low-level oil exposure also compromises immunological function, reproductive success, and offspring survival in affected populations [63,109]. Because many seabirds travel widely and feed high in marine food webs, their contaminant burdens integrate PAH exposure over broad ocean areas, so their health condition provides a useful proxy for ecosystem-scale pollution patterns [110]. For example, loons wintering in South Korea showed elevated BaP-equivalent DNA damage biomarkers attributed to continued trophic exposure to petrogenic PAH sources. It indicates that sentinel monitoring of migratory seabirds can provide indicators of pollution trends across vast oceanic areas [110]. Similarly, deer in peri-urban and industrial zones in Central Europe have been proposed as terrestrial mammalian sentinels, with studies detecting PAH-related biomarkers across tissues of roe deer and red deer spanning areas of differing industrialization [98].

#### 3.3.3. Wildlife as Sentinels for Human Health

The value of wildlife as biological sentinels for PAH contamination rests on several complementary features. First, fish, marine mammals, and bivalves integrate PAH contamination over time in their tissues and bile, providing spatially resolved, time-averaged records of environmental contamination that supplement point-in-time water and sediment measurements [51,58]. Second, the hepatic pathological changes and DNA adducts found in field-collected fish constitute biomarkers of biologically effective dose, providing evidence that PAH contamination has not merely entered the environment but has interacted with biological macromolecules in ways relevant to disease, providing a bridge between environmental monitoring and health risk assessment [60,103]. Third, the apex marine mammals and raptors that share food webs with human seafood consumers experience PAH exposures from the same contaminated prey, making their health status a biologically informed indicator of potential human dietary risk from those food sources [111].

## 4. Discussion

### 4.1. One Health Integration: Connecting the Three Domains

The One Health framework provides essential conceptual architecture for interpreting the complex, interconnected challenge posed by PAH contamination. Unlike many classic persistent organic pollutants that mainly pose risks through biomagnification in fatty tissues, PAHs represent a One Health concern driven by their near-ubiquitous release from combustion, ranging from industrial fuel use to household cooking, as well as by the highly conserved cellular pathways through which they form DNA-reactive metabolites and cause genotoxicity across species [8,16,28]. Together, these two features ensure that PAH exposure and its health consequences are shared across the full spectrum of organisms inhabiting combustion-dominated environments.

The food web, although not amplifying PAH concentrations in the manner of organochlorine pesticides or PCBs for example, constitutes a critical conduit linking contaminated aquatic sediments to the dietary exposure of both wildlife predators and human consumers of fish and seafood. The key integration point is the benthic–pelagic interface of coastal and freshwater systems. PAHs deposited from the atmosphere and transported by urban runoff accumulate in sediments, are bioconcentrated by benthic invertebrates with limited metabolic capacity, being then transferred upward through the food web to commercially important fish species, ultimately reaching human plates [51,54,112]. In this pathway, the health of the benthic ecosystem (the diversity and abundance of the invertebrate community, the quality of the sediment habitat) is directly relevant to human dietary risk, a One Health linkage too rarely articulated in food safety risk assessment.

The remarkable consistency of toxicological effects across vertebrate species, particularly hepatotoxicity, genotoxicity, and reproductive impairment mediated through AhR-dependent pathways, validates the use of fish and wildlife data to assess human health risk and vice versa [6,21]. Reports of elevated cancer rates in beluga whales from the St. Lawrence Estuary and liver tumors in English sole from Puget Sound have been systematically documented in these wildlife populations, and the underlying PAH contamination in nearby human populations raises analogous concerns. It shows how PAH-associated malignancies in wildlife could serve as an early warning signal for cancer risks in coastal communities within a One Health surveillance context [62,103].

An important complexity in the One Health PAH integration concerns mixture toxicology. Environmental PAH contamination invariably involves mixtures of dozens to hundreds of individual congeners, co-occurring with other combustion-derived contaminants, including particulate matter, organometallic compounds, and volatile organic compounds [2,113]. The conventional method of assessing PAH risk through BaP equivalents based on toxic equivalency factors (TEFs) accounts for part of the mixture’s complexity. However, current TEF frameworks are limited to a few well-characterized congeners and rely on the assumption of additive toxicity, which may overlook possible synergistic or antagonistic interactions among compounds in environmental mixtures [7]. This is a critical scientific and regulatory gap that applies equally to wildlife risk assessment and human risk assessment, linking them through a common methodological challenge. Specific examples of interactions between PAHs and co-occurring pollutants with One Health implications include the potentiation of PAH-induced immunosuppression by co-exposure to organochlorine pesticides such as endosulfan and chlorpyrifos in aquatic organisms [56], and the modulation of AhR-mediated induction by co-exposure to alkylated PAH congeners derived from petroleum hydrocarbon releases [64].

### 4.2. Knowledge Gaps and Research Needs

In the context of One Health research, a major challenge in PAH science is to elucidate the complex trophodynamic behavior of these compounds across aquatic and terrestrial ecosystems. While parent PAHs often display trophic dilution, this trend can obscure selective retention of hydrophobic congeners, restricted metabolic processing in certain species, tissue-specific accumulation patterns, and the trophic transfer of biologically active metabolites. The evidence for biomagnification remains mixed, as many studies are limited to a subset of congeners, overlook high-molecular-weight PAHs (>302 Da) [42] and substituted analogues, or inadequately differentiate parental structures from transformation products [53,54]. To advance understanding, future investigations should integrate congener-specific trophic analysis with stable isotope approaches, lipid normalization, metabolite profiling, and evaluation of species-dependent metabolic capacity. Expanding these frameworks to include terrestrial indicators such as domestic animals and peri-urban wildlife would significantly improve the ecological and translational scope of One Health PAH assessment.

Substantial knowledge gaps remain, which impede fully integrated One Health management of PAH contamination [26,99,114]. These gaps span multiple domains and scales, from the molecular mechanisms of mixture toxicity to the landscape-level consequences of climate-driven changes in wildfire frequency and distribution. To provide a concise overview of these outstanding issues, Table 2 summarizes the major research gaps in PAH toxicology, trophodynamics, climate interactions, biomonitoring equity, and wildlife/ecosystem impacts, together with their key One Health implications.

Developing mixture toxicology frameworks that can characterize how PAHs interact with co-occurring pollutants, such as particulate matter, organometallic compounds, PCBs, and emerging contaminants, across human and wildlife exposure scenarios is a research priority. In contaminated environments, PAHs are virtually never encountered as single compounds [115]. However, most experimental toxicology still focuses on isolated PAHs, reporting data with limited direct relevance for the complex exposure patterns that prevail in real-world settings [116].

The trophodynamics of PAH transfer in marine and freshwater food webs require further investigation, particularly regarding the role of species-specific differences in CYP1A expression and activity in modulating biologically effective dose across trophic levels [54]. The apparent contradiction between trophic dilution of parent PAH compounds and the potential for trophic transfer of reactive PAH metabolites, which may themselves be biologically active, has not been adequately resolved and has important implications for food safety risk assessment [53,54].

Climate change interactions with PAH cycling represent an emerging research priority of considerable One Health significance [117,118]. Increases in wildfire frequency and intensity projected under warming scenarios would substantially augment atmospheric PAH loading, depositing these contaminants across vast terrestrial and aquatic ecosystems currently experiencing relatively low PAH burdens [11,35]. Therefore, the consequences for wildlife populations and indigenous communities relying on traditionally uncontaminated food resources from these ecosystems require urgent investigation.

Achieving global equity in biomonitoring remains a critical gap in current research. The preponderance of PAH data originates from America, Western Europe, and East Asia, with limited information from Africa and South and Southeast Asia, as well as from most countries of Latin America, regions characterized by high rates of solid fuel combustion for cooking and heating, extensive open biomass burning, and rapidly growing industrial sectors [2,9,67,93]. Without representative biomonitoring data from these regions, risk assessments are systematically anchored in populations that already benefit from the strongest regulatory protections, thereby reinforcing the under-recognition of communities bearing the highest PAH exposure burdens in global risk governance (Table 2). Indoor air data scarcity and insufficient biomonitoring of nursing mothers and school-aged children in these underrepresented regions are particularly notable sub-gaps within this broader equity challenge.

### 4.3. Policy Implications and Intervention Strategies

The complex character of PAH pollution calls for interventions that concurrently target emission sources, exposure pathways, and associated health effects within a One Health framework encompassing humans, animals, and the environment. From this perspective, the most impactful and far-reaching measure is still source reduction via improved combustion control, which includes shifting toward cleaner energy systems, tightening and enforcing vehicle emission standards, enhancing industrial combustion efficiency, and curbing open biomass burning can collectively lower PAH levels in air, soil, water, and food while also yielding co-benefits for respiratory health, wildlife habitat quality, and overall ecosystem integrity [2,9,42]. An integrated overview of One Health-oriented intervention strategies for PAH management, linking concrete actions (from source control to occupational protection) to cross-domain co-benefits and implementation challenges is shown in Table 3.

Dietary PAH exposure, dominated by high-temperature cooking methods, represents a behavioral pathway suitable for targeted intervention without requiring systemic industrial change [119]. Evidence-based cooking advisories, recommending reduced charbroiling temperatures, removal of charred food surfaces, increased use of marination which has been shown to reduce PAH formation, and alternatives to direct flame contact, can reduce the primary PAH exposure route for most consumers [37,120,121]. Harmonized international maximum levels for PAHs in smoked and grilled food products, combined with improved enforcement capacity in low- and middle-income countries, would extend these protections to the most highly exposed dietary populations.

Legacy contaminated sites (coal gasification plant residues, former tar production sites, contaminated harbor sediments) are persistent PAH reservoirs that require active remediation. Bioremediation using PAH-degrading microbial consortia has shown promising results at pilot scale, offering a cost-effective approach for surface soils [122]. Nevertheless, its efficacy diminishes with increasing molecular weight and decreasing PAH bioavailability [47]. Integrated monitoring programs linking environmental PAH surveillance with wildlife biomonitoring and human urinary metabolite biomonitoring, following principles analogous to those proposed for other environmental pollutants [30,31,32], would enable early detection of emerging exposure concerns and provide evidence for adaptive management.

Environmental justice principles should be explicitly incorporated into policies addressing PAH exposures, as vulnerable communities, which are often located near industrial sites, reliant on solid fuel combustion for cooking, or consuming fish from contaminated waters, face disproportionate health risks [91,92,97]. Policy frameworks that apply cumulative risk assessment to PAH exposures, accounting for co-occurring chemical and social stressors, and institutionalize meaningful community participation in contaminated site decisions, are critical to achieving equitable health protection [91]. With respect to Table 3, “strengthened standards” in the occupational protection row refers specifically to the adoption and harmonized enforcement of Biological Exposure Indices (BEI) for PAH biomarkers (particularly 1-OHP and PAH-DNA adducts) across jurisdictions, combined with regular review and updating of occupational exposure limits (OELs) for individual PAH compounds.

## 5. Conclusions

Polycyclic aromatic hydrocarbons (PAHs) represent a persistent, globally distributed, and toxicologically consequential class of environmental pollutants that exemplify the need for a unified One Health approach to risk assessment and management. Their widespread occurrence, arising from combustion-based energy systems, food processing, and natural landscape dynamics, creates overlapping exposure environments for humans, domestic animals, and wildlife across nearly all ecosystems. This contamination highlights the inextricable links between environmental pollution, ecosystem integrity, and human health.

The coupling between environmental PAH contamination and biological exposure is most evident in aquatic systems, where sediments serve as long-term reservoirs. Benthic invertebrates accumulate parent compounds, while fish metabolize and bioactivate PAHs into reactive intermediates. This sediment–invertebrate–fish axis forms both a mechanistic and ecological bridge connecting ecosystem processes to population-level health outcomes in wildlife and humans. The evolutionary conservation of the aryl hydrocarbon receptor (AhR) pathway further strengthens this link, as the same genotoxic cascades driving PAH-induced carcinogenesis in aquatic organisms are operative in human populations exposed through air, diet, or occupational settings. This mechanistic convergence supports the use of wildlife as sentinels of environmental contamination and highlights the shared biological vulnerability across species.

Adverse health effects documented in wildlife (hepatic neoplasms in demersal fish, reproductive and developmental anomalies in marine mammals, and immunotoxicity in seabirds) provide biologically anchored evidence of harmful exposure and serve as early-warning indicators relevant to human health risk assessment. However, PAH exposure is unevenly distributed across human populations. Elevated exposures are observed among children, pregnant women, workers in combustion-intensive sectors, and communities dependent on contaminated food sources or situated near industrial emissions. These disparities introduce clear environmental justice dimensions that must be integrated into public health policy and regulatory actions.

Crucially, current challenges extend beyond the traditional set of priority PAHs. Emerging research indicates that high-molecular-weight PAHs (>302 Da), alkylated homologues, and diverse transformation products may contribute disproportionately to environmental persistence, selective trophic transfer, and toxic potency, yet remain underrepresented in monitoring efforts and regulatory frameworks. Incorporating these underexplored PAH classes within a One Health framework is essential for capturing the full scope of combustion-derived contamination and its ecological and human health implications.

Despite decades of investigation, major knowledge gaps persist in mixture toxicology, food-web trophodynamics, interactions between climate processes and contaminant behavior, and equitable global biomonitoring. Addressing these gaps demands coordinated, interdisciplinary research linking toxicology, ecology, environmental chemistry, epidemiology, and public health governance. Only through such integrative approaches can the multifaceted nature of PAH contamination be accurately characterized and mitigated.

Strategically, the advancement of One Health PAH science should converge along three main axes: (i) expansion beyond legacy priority PAHs, (ii) mechanistically informed research on trophic transfer and biomagnification, and (iii) harmonized surveillance across environmental, animal, food, and human matrices. Implementing such a framework is vital for transforming descriptive contamination profiles into predictive, prevention-oriented tools for public health and ecosystem protection. Future surveillance systems should integrate environmental monitoring, sentinel species studies, food-web analysis, and coordinated human biomonitoring while accounting for cumulative exposure, climate-driven redistribution, and social equity considerations.

Viewed through a One Health lens, challenges as varied as urban air pollution, seafood safety, wildlife population decline, and occupational cancer risk reflect facets of a single interconnected problem with shared drivers and solutions. Therefore, effective mitigation of PAH contamination requires recognition of these interdependencies and the adoption of collaborative scientific, regulatory, and public health strategies, which safeguard ecosystems, wildlife, and human communities alike.

## Figures and Tables

**Table 1:** Major sources, exposure pathways, and adverse effects of PAHs across One Health domains.

Domain	Primary Sources	Exposure Pathways	Adverse Effects
Environmental	Fossil fuel combustion (vehicular exhaust, power generation); Biomass burning (wildfires, agricultural burning); Industrial processes (coking, aluminum smelting, petroleum refining); Oil spills and runoff; Residential heating (wood and coal burning)	Atmospheric deposition to soil, water, and vegetation; Stormwater and urban runoff; Sediment accumulation in aquatic environments; Long-range atmospheric transport	Persistent soil and sediment contamination; Ecosystem disruption and degradation; Alteration of microbial communities; Phytotoxicity in contaminated soils; Aquatic ecosystem impairment
Wildlife/Animal	Contaminated aquatic sediments; Atmospheric deposition on terrestrial habitats; Food web bioaccumulation in lower trophic species; Oil spills (marine and freshwater)	Dietary intake of contaminated prey; Direct contact with contaminated water/sediment; Dermal absorption (aquatic species); Maternal transfer (eggs, placenta, lactation); Inhalation near combustion sources	Immunosuppression; Genotoxicity and carcinogenesis; Reproductive and developmental toxicity; Endocrine disruption (AhR-mediated); Hepatotoxicity; Population decline in sensitive species
Human	Grilled, smoked, and charbroiled foods; Air pollution (urban and indoor combustion); Occupational exposures (coke ovens, firefighting, asphalt); Contaminated soil near industrial sites; Drinking water (near contaminated groundwater)	Dietary intake (primary route); Inhalation of ambient and indoor air; Dermal absorption; Occupational inhalation and dermal contact; Maternal-fetal transfer and breast milk	Lung cancer and other malignancies; Genotoxicity (DNA adducts via CYP1A1/1B1 activation); Immunotoxicity; Reproductive and developmental toxicity; Cardiovascular effects; Neurodevelopmental effects in children
Key Interconnections	Food webs create direct linkage: environmental contamination → benthic organisms → aquatic consumers → human dietary exposure; Shared AhR-mediated toxicity produces convergent carcinogenic and immunotoxic effects across species; Wildlife (especially fish and marine mammals) serve as sentinels of PAH contamination predating human health impacts; High-temperature combustion links air quality, wildlife inhalation exposure, and human respiratory risk simultaneously		

All information in this table is supported by the references cited in the corresponding sections of the main text.

**Table 2:** Critical knowledge gaps and research priorities for One Health PAH management.

Research Area	Critical Knowledge Gaps	One Health Implications and Priorities
Toxicology and Mixture Risk Assessment	Poor characterization of interactive effects; limitations of single-compound models; non-linear dose–response uncertainties.	Develop realistic mixture models; cross-species integration for risk characterization.
Long-term and Multigenerational Effects	Limited longitudinal data; unclear epigenetic mechanisms; insufficient developmental data.	Establish long-term cohorts; clarify lifetime risks.
Trophodynamics and Bioaccumulation	Contradictory trophic transfer findings; metabolism uncertainties; limited congener data.	Conduct congener-specific food web studies.
Climate Change Interactions	Uncertain wildfire impacts; volatilization changes; marine partitioning gaps.	Model climate–PAH scenarios for adaptive management.
Global Biomonitoring and Environmental Justice	Regional data gaps; insufficient vulnerable population biomonitoring; indoor air data scarcity; limited data on nursing mothers and school children in LMICs.	Expand harmonized global biomonitoring; prioritize nursing mothers, infants, and children in under-monitored regions.
Wildlife Population and Ecosystem Impacts	Limited demographic linkage; understudied cumulative stressors; scarce terrestrial monitoring.	Link exposure to population outcomes; strengthen sentinel systems.

**Table 3:** Integrated One Health intervention strategies for PAH management.

Intervention Category	Specific Strategies	Cross-Domain Co-Benefits	Implementation Challenges
Source Control and Emission Reduction	Stricter vehicle emission standards; transitioning to cleaner energy; industrial BAT; regulation of biomass burning; improved heating technologies.	Environmental, wildlife, and human co-benefits through reduced deposition and exposure; co-reduction in co-emitted pollutants.	Economic costs; technological gaps; enforcement limitations; energy transition constraints.
Dietary Exposure Reduction	Cooking advisories; maximum residue levels; improved smoked food standards; dietary guidance for vulnerable groups.	Direct reduction in dietary exposure; awareness raising.	Cultural practices; regulatory variability.
Remediation of Contaminated Sites	Bioremediation; phytoremediation; chemical oxidation; sediment capping/dredging; monitored natural attenuation.	Habitat restoration; reduced benthic exposure; community protection.	High costs; technical limitations; redistribution risks.
Integrated Surveillance and Monitoring	Environmental monitoring; wildlife biomonitoring; human biomonitoring; food monitoring.	Early detection; sentinel systems; targeted intervention.	High analytical costs; harmonization needs; sustained funding.
Occupational Protection	Enhanced PPE; engineering controls; biomonitoring (1-OHP, PAH-DNA adducts); strengthened occupational exposure limits (OELs) and biological exposure indices (BEIs) reviewed against current toxicological evidence.	Protection of high-risk workers; reduced emissions.	Enforcement variability; cost burdens.
Regulatory Frameworks	Harmonized standards; mixture inclusion; transboundary cooperation; environmental justice integration.	Coordinated global protection across domains.	Jurisdictional complexity; political constraints.

## Data Availability

No new data were created or analyzed in this work. Data sharing is not applicable to this article.
